# *Jitegemee* (rely on yourself): a multi-phase process of co-creating a personal savings intervention with female sex workers in western Kenya to reduce their HIV risk

**DOI:** 10.1186/s12889-024-20348-5

**Published:** 2024-10-18

**Authors:** Kawango Agot, Jacob Onyango, Marylyn Ochillo, Timothy Omondi Okello, Shantana Carol, Tobias Odwar, Jane Moraa, Sophie Otticha, Redempter Odeny, Nicky Okeyo, Linet Ochieng, Gerald Ochieng, Ivy Wango, Alloys Koloo, Jacinta Badia, Carol S. Camlin, Bernard Ayieko, Sue Napierala, Harsha Thirumurthy

**Affiliations:** 1 Impact Research and Development Organization, P.O Box 9171-40141, Kisumu, Kenya; 2 Masinde Muliro University of Science and Technology, Kakamega, Kenya; 3 Kenya Medical Research Institute, Kisumu, Kenya; 4 Department of Obstetrics and Reproductive Sciences, University of California, San Francisco, USA; 5 Department of Medicine, Division of Preventive Sciences, University of California, San Francisco, USA; 6 Women’s Global Health Imperative, RTI International, Berkeley, CA USA; 7 Department of Medical Ethics and Health Policy, University of Pennsylvania, Philadelphia, USA

**Keywords:** Female sex workers, Co-design, Savings intervention, Economic empowerment, Sustainability

## Abstract

**Background:**

HIV prevalence among female sex workers (FSW) is significantly higher than among women in the general population. Studies have shown that FSW engage in unprotected sex which provides higher compensation when they face emergency situations. We co-created a savings intervention – *Jitegemee* (rely on yourself) – with FSW to encourage them to save part of their earnings to withdraw in emergency situations in order to reduce risk.

**Methods:**

We undertook a five-phase intervention development process between February 2021 and July 2023: 1) qualitative interviews with FSW to identify essential intervention features; 2) pilot trial to assess intervention feasibility; 3) literature review of studies on economic empowerment of FSW; 4) scoring of key components of Phases 1–3 on a scale of 1–5 (1 = definitely exclude, 5 = definitely include), for inclusion in the intervention package; 5) workshops with FSW and other key stakeholders to co-design the intervention.

**Results:**

In phase 1, nearly all participants (99%) found the intervention acceptable to them and 95% believed it would be acceptable to other FSW. Participants suggested inclusion of financial literacy (75%), savings groups (38%) and goal-setting (24%). In the feasibility assessment, 41% saved, of whom 46% withdrew some savings. Condom use was higher among FSW who withdrew their savings compared to those who did not (χ^**2**^ 7.52; *p* = 0.006). In Phase 3, we identified 14 intervention components. In phase 4, all suggested intervention components scored 4.5 on average. In phase 5, we held 3 workshops with FSW to co-design the intervention, which included instructions for how to save and make withdrawals, financial literacy training, and formation of savings groups.

**Conclusions:**

A savings intervention for and by FSW was highly acceptable and feasible. Involving end-users in the design process is likely to result in greater economic security among FSW and lower engagement in higher risk transactional sex.

## Background

Female sex workers (FSW) comprise only 1.1% of women of childbearing age [1] yet contribute 12% of the HIV infections globally [2]. In Kenya, HIV prevalence among FSW is about 5-times higher than that of women in the general population [3]. Relationship power between FSW and their male clients is imbalanced [4–6], and FSW, especially in poorer settings, have low agency to insist on condom use [7] or decline a higher pay that comes with condomless sex [8] or anal sex [9, 10]. Reaching the Joint United Nations Programme on HIV/AIDS (UNAIDS) goal of ending the AIDS epidemic by 2030 [11] will remain elusive if effective HIV prevention interventions among FSW do not boost their economic security in ways that embolden them to decline sex with certain clients or to negotiate for safer sex. HIV prevention interventions for FSW have identified economic factors that put them at elevated risk including financial insecurity occasioned by poor economic status, lack of control over resources, scarcity of alternative livelihood options, financial debts, and food insecurity [12–14].

Economic empowerment of FSW is an enabling strategy that can FSW make important decisions that determine their lives [12, 15] including their choice of work and their ability to reduce their HIV vulnerability. A study among FSW in Kenya on precautionary savings in a mobile banking platform showed a majority adopting the mobile savings and a reduction of risky sexual behaviours with the introduction of cell phone-based saving programmes [13]. In Kazakhstan, FSW who participated in the Nova intervention, which had a savings component, reported strong interest in a matched-savings intervention [16]. The Usha Multi-Purpose Co-operative Society intervention programme implemented in South Africa improved economic status among FSW by increasing savings and reducing the economic vulnerability that can affect their capacity to negotiate condom use with clients [17].

An outstanding aspect that is missing in most of these intervention is that they do not fully address sexual encounters that arise from desperation following economic shocks that result in lower client flow and/or higher need for money from unprotected sex. Studies have shown that such economic shocks are extremely common in resource-limited settings in Sub-Saharan Africa (SSA), stemming from developments such as weather events, illnesses in the family, or unemployment [13, 18, 19]. Helping FSW cope with these shocks in ways that do not increase their HIV risk is essential for reducing new infections in this priority population. Identifying effective strategies to address economic disempowerment that puts FSW at ongoing HIV risk is thus an important policy priority [20, 21].

At the core of such interventions would be coming up with what FSW would accept and use, and one way to achieve that is to work together with representatives of the end-user population and other stakeholders to co-create the intervention. Engagement of potential end-users in participatory action research (PAR) and co-designing of interventions is rapidly gaining traction [22–25]. The likelihood that research results will be relevant to and valued by end-users and their communities is enhanced when the perspectives of the researchers and the target population(s) jointly inform the design of the intervention products [26]. Jun and colleagues urge researchers to explicitly seek views and voices of stakeholders involved in, and impacted by, the intervention at the key moments of the intervention design process to assure that not only are the outcomes of the intervention effective, but the processes to achieve the results are not ethically objectionable [27]. It is therefore prudent to bring end-users on board earlier in the intervention design stage, to partner with researchers in developing interventions that would be more acceptable and adhered to when rolled out [26, 28].

Multiple studies have been conducted in Kenya and elsewhere on economic empowerment of FSW and most are often geared towards the ‘rehabilitation’ of FSW and prepare them to leave sex work altogether [29–31] instead of identifying economic factors that put them at elevated risk and intervening to reduce risk while practicing sex work [13, 32–36] and premised on the assumption that economic hardship drives women into sex work, therefore providing alternative source of income would draw them away from the sex trade [37]. Such approaches may not be very effective because some FSW may experience challenges transitioning to alternative income due to unresolved socio-economic, health, and other challenges [16]. However, focusing on securing alternative income while focusing on the role of savings and financial management can promote financial security which may reduce the urgency of sex work and their need to engage in unprotected sex with clients in order to earn more [12, 38, 39].

A study by Jones and Gong [13] was piloted in western Kenya, whereby researchers enrolled vulnerable women including FSW and encouraged them to save on MShwari – a mobile money saving platform in Kenya. They found the mobile savings intervention successful, with a 60% participant adoption of the intervention. While this is promising, it is important to develop a savings intervention that will impact sexual risk-taking, and explore how savings can ensure financial security in the short- and long-term. This paper chronicles the steps taken to co-design a savings intervention with FSW as end-users, civil society organization for FSW, implementers of FSW programs, and policy makers from the Ministry of Health (MOH) in three counties in western Kenya.

## Methods

We undertook a 5-phase co-design process to come up with an intervention – known as *Jitegemee* (Kiswahili for ‘rely on yourself’) – that aims to encourage FSW to save part of their earnings for use when faced with immediate financial need or economic shock that might compel them to engage in risky sex. The phases were: 1) acceptability, 2) pilot feasibility, 3) in-depth review of published literature, 4) scoring and prioritizing preferred components to include in the intervention package, and 5) holding workshops to design the intervention using information obtained from phases 1–4.

### Phase 1 – acceptability

Between February and April 2022, we conducted a mixed methods study comprising of quantitative and qualitative data collection with FSW in Kisumu and Siaya counties. We enrolled FSW representing key typologies (venue-, street-, home-, brothel-, and beach-based) and asked them questions about their saving, spending, borrowing and lending practices; whether they would accept an intervention that requires them to save part of their earnings for use in emergency situations; components to include in the intervention package; and strategies they would employ to obtain money to save. Before being asked questions on their views about the proposed intervention, it was explained to the FSW that the *Jitegemee* intervention would be anchored on the belief that if supported, they would plan to save part of their earnings to reach a certain level of economic security that allows them to say no to unsafe sex without fear of losing income for basic needs. Those who would wish to quit sex work were informed that the intervention would involve asking them their preferred path to economic independence during or after sex work, how they can save towards their goals and how long it would take to reach those goals, then support them to set realistic goals and timelines, and to work towards achieving them.

### Phase 2 – pilot randomized-controlled feasibility study

Between March 2022 and April 2023, we conducted a randomized-controlled pilot feasibility study (known as *Saving for a Rainy Day*) in Siaya County to assess if FSW would actually save part of their earnings, if they would withdraw their savings when in need of cash, and whether withdrawing money would translate to safer sex practices, particularly condom use. A detailed description of the methods has been reported elsewhere (Ochieng’ LA, Onyango J, Owuor GO, Obare I, Bukusi E, Agot K: Saving for a rainy day: A randomized-controlled trial to pilot a savings intervention to reduce HIV risk among young female sex workers in Siaya County, Kenya, submitted), but briefly, FSW in the intervention arm were given instructions on how to save through a mobile saving platform – Mpesa – that transferred funds to a designated study-run bank account, and how to withdraw their savings whenever they need money back. Women in the control arm received no intervention. At month six, we administered a quantitative questionnaire, measuring reported saving and condom use; we also assessed savings and withdrawal behaviors of intervention participants through bank records.

### Phase 3 – in-depth review of published literature

In order to make the design applicable to FSW in other settings, two co-authors (JO, MO) searched PubMed and Google Scholar for literature on studies conducted among FSW in Africa and Asia articles. The literature review focused on articles published over a 20-year period, between 2003–2023, based on studies that involved the following economic interventions: individual or group savings, microfinance, financial literacy training, vocational training, micro-enterprises, and asset ownership. Studies included in our review assessed the impact of the interventions (individually or a combination) on sexually transmitted infection (STI) and/or HIV risk behavior among FSW.

### Phase 4 – scoring

We compiled a list of all components of economic empowerment interventions derived from phases 1–3, and listed a total of 19 stand-alone components. Between June and August 2023, we presented the list to FSW in Siaya and Homabay counties and asked them to score how important each component was for inclusion in a savings intervention that could be tested on a larger scale. The scoring was done on a scale of 1–5 (1 = definitely exclude to 5 = definitely include). To ensure the proposed intervention would be sustainable, participants were also asked to score each component in terms of whether it would require external financial or logistical support to implement (1 = definitely requires outside resources to 5 = definitely does not require outside resources). After initial analysis showed that participants scored most items 4 and 5, they were asked via phone to prioritize their top five components into the most to least preferred (5 = most; 1 = least) for inclusion in the intervention. Finally, participants were asked to list potential shocks that might affect FSW’s ability to save, and the likelihood of the shocks occurring in the foreseeable future, defined as the next 6 months. An average of the total weighted score was then calculated for each intervention component to determine those with the highest ranking to consider for inclusion in the intervention package.

### Phase 5 – designing the intervention

After identifying the top 5 intervention components, we convened three co-design workshops. All three started with an overview of what *Jitegemee* intervention hopes to achieve, the studies already conducted to obtain information to inform the design, and the key findings. The first workshop was held with FSW peer educators (PE) who had recruited participants for phases 1, 2 and 4. A peer education approach to HIV programming is a strategy where key populations such as FSW nominate their trusted peers to support the delivery of HIV and other interventions [40]. Through iterative discussions, the PE and key study staff developed the first draft of the intervention.

The second workshop brought together representatives of a civil society organization overseeing thousands of sex workers in western Kenya, and development partners implementing HIV programs with key populations including FSW. In addition to reviewing and modifying the draft intervention package developed during workshop 1, the team also suggested possible implementation strategies.

The last workshop was held with MOH senior personnel who make policy decisions and guide HIV program implementation in the three counties where phases 1, 2 and 4 were conducted. Specifically, discussants included representatives from the key population technical working group in each county, county AIDS and STI coordinating offices, and members of the county and sub-county health management teams. Their primary role was to review the revised, near-final, intervention package and make policy-relevant recommendations.

## Results

### Phase 1: acceptability

Results of the acceptability phase have been reported elsewhere [10, 41]. Briefly, of the 369 FSW enrolled in Kisumu and Siaya counties, 98.9% and 94.8% found the intervention acceptable for themselves and for other FSW in Kenya, respectively. They suggested saving literacy, sharing best saving practices and challenges by peers, goal setting, and training on loan management, among others, as important components they would like to see in the intervention package. In the qualitative sub-study, 221 FSW were engaged in 24 focus group discussions (FGD). They also found personal saving interventions acceptable and added that the *Jitegemee* intervention should equip FSW with knowledge and skills on how to balance earnings, spending, savings, and taking and repaying loans.

### Phase 2 – a pilot feasibility study

A total of 207 FSW were enrolled and randomized to the intervention and control groups. Results have been reported (Ochieng’ LA, Onyango J, Owuor GO, Obare I, Bukusi E, Agot K: Saving for a rainy day: A randomized-controlled trial to pilot a savings intervention to reduce HIV risk among young female sex workers in Siaya County, Kenya, submitted), but in summary, 40.6% of intervention arm participants saved, of whom 46.3% withdrew part or all of their money. The key risk behavior indicator assessed (reported condom use) was significantly higher among intervention participants who saved and withdrew their money compared to those who saved but did not withdraw their money (χ^**2**^ 7.52; *p* = 0.006).

### Phase 3 – literature review

We reviewed 13 randomized trials, 4 qualitative studies (including in-depth interviews, FGDs and an observational study), 1 formative mixed method study, 1 pre-post survey study and 1 quasi-experimental study across 8 countries, 5 in SSA (Kenya, Uganda, Tanzania, Côte d’Ivoire and South Africa) and 3 in Asia (India, Mongolia and Kazakhstan). Overall, we abstracted 68 intervention components from the 20 studies, of which 14 were unique, including financial literacy training, with components such as savings, budgeting, debt management, and financial negotiation; business skills training; asset ownership; micro-enterprise; vocational training; assessing of income and expenditure; and financial management. The 14 intervention components were added to the list of interventions scored and prioritized by participants for inclusion in the Jitegemee intervention package (Table 1). Of the 16 studies that assessed the impact of the intervention on HIV/STI risk, 12 [12, 13, 42–49] were effective, and 4 [16, 50–52] did not report results on HIV/STI risk.

**Table 1:** Scoring and prioritization of suggested components for inclusion in the Jitegemee intervention package (*N* = 185)

Intervention components	Scoring Mean	n (Ranking Points)					Ranking Average points
		**Preference1**	**Preference2**	**Preference3**	**Preference4**	**Preference5**	
Educational Support	4.83	33(165)	35(140)	27(81)	12(24)	11(11)	84.2
Asset Transfer	4.47	16(80)	16(64)	18(54)	10(20)	12(12)	46
Micro Insurance	4.5	10(50)	15(60)	18(54)	15(30)	15(15)	41.8
Micro credit	4.48	10(50)	19(76)	13(39)	12(24)	11(11)	40
Unconditional Cash Transfers (UCTs)	4.39	22(110)	10(40)	6(18)	9(18)	7(7)	38.6
Vocationalor entrepreneurial training	4.77	8(40)	14(56)	16(48)	17(34)	14(14)	38.4
Business development trainings	4.68	7(35)	14(56)	14(42)	16(32)	11(11)	35.2
Conditional Cash Transfers (CCTs)	4.4	20(100)	4(16)	10(30)	7(14)	7(7)	33.4
Savings Buddy Groups	Not scored	8(40)	4(16)	11(33)	12(24)	17(17)	26
Financial capabilities education/Training	4.7	5(25)	10(40)	7(21)	15(30)	11(11)	25.4
Community savings groups	4.39	10(50)	6(24)	8(24)	6(12)	10(10)	24
Income generation	4.56	8(40)	9(36)	5(15)	7(14)	9(9)	22.8
Transportation Assistance	4.38	5(25)	4(16)	8(24)	6(12)	12(12)	17.8
Financial Incentives	4.32	7(35)	4(16)	5(15)	8(16)	4(4)	17.2
Employment Support	4.57	5(25)	6(24)	5(15)	7(14)	6(6)	16.8
Food Aid / Assistance	4.68	1(5)	5(20)	5(15)	13(26)	17(17)	16.6
Financial Literacy Training	4.68	3(15)	3(12)	4(12)	3(6)	3(3)	9.6
Asset ownership	4.31	1(5)	3(12)	2(6)	2(4)	2(2)	5.8
Debt Management	4.43	1(5)	2(8)	1(3)	3(6)	3(3)	5

### Phase 4 – scoring exercise

We presented the list of 19 intervention components to 250 FSW from Homabay and Siaya counties. Their average score was 4.5 on a 5-point scale for all components (1 = definitely exclude, 5 = definitely include). Due to the high scores of the proposed components, 185 participants were re-contacted via phone and asked to rank their top 5 components which they had scored 4 (consider including) or 5 (definitely include) in order of priority. Both scoring and prioritization results are presented in Table 1 (savings buddy groups was inadvertently omitted from the scoring template hence not scored; however, since it was suggested in phase one and all components scored between 4 and 5, we included it in the ranking exercise).

Finally, the top three shocks that participants in phase 4 felt could affect their ability to save were the rapidly rising cost of living in Kenya (67.6%), sickness (65.6%), and low male client flow (56.4%).

### Phase 5 – intervention design

#### Design workshop 1

Eighteen PEs from the Kisumu, Siaya and Homabay counties and five research team members participated in the first workshop that took two full days. We presented results from Table 1 and brainstormed over each suggested component. While there were strong opinions over inclusion of most components that scored 4 and 5 and ranked top 1 and 2, the following high-scoring and high-ranking components were ruled out due to their reliance on external funding, making them unsustainable: educational support, unconditional and conditional cash transfer, asset transfer, micro credit and insurance. Participants also observed that some FSW are already at different saving levels and therefore the goal of *Jitegemee* should not only be to create a financial reserve for withdrawal when in need but to also consider incorporating saving for short- or long-term investments. Consequently, the team set two saving tiers (Tier 1 for withdrawal, set at a maximum of KShs. 2,000 (≈US$13) was considered for call back when a FSW was faced with emergency situations that require immediate cash such as fare back home and other basic household expenses including schooling materials for children, sanitary pads and medical bills. This was estimated to be equivalent to the average earning for a day that may sustain a FSW for a while. The amount for and Tier 2 was set aside for short and/or long-term goals, comprising of any amount saved beyond the maximum for Tier 1). The team designed the first draft of the intervention package, comprised of financial literacy training (budget planning and tracking income, expenditure and loans), goal setting, saving buddy groups to share saving challenges and best practices (through WhatsApp or short messaging system), and subscription to the National Hospital Insurance Fund (NHIF) as security over emergency illness of self or family.

#### Design workshop 2

Seven stakeholders (from 4 implementing partner organizations and one civil society organization for FSW) and five research team members participated in a full day workshop. After providing stakeholders with the background of *Jitegemee* and the key results of phases 1–4, we presented the first draft intervention package developed during workshop 1, for their input. They expressed reservations about inclusion of NHIF as it would require KShs. 500 be deducted from FSW’s savings and remitted to the Fund monthly, which they felt may be too high and discourage some from saving. However, because it was suggested by PE, the team was hesitant about dropping it. The team pointed out that the intervention components should address structural, behavioral and biomedical determinants of HIV risk among FSW. They recommended that during implementation, attention should also be paid to strengthening social support systems for FSW, addressing their alcohol and drug use, and supporting response to sexual and gender-based violence.

#### Design workshop 3

A total of 15 stakeholders from the MOH in the three counties and eight research team members participated in the final half-day workshop. As in workshop 2, participants were taken through the goal of the *Jitegemee* intervention, highlights of phases 1–4, and the design processes and products of workshops 1 and 2. They applauded the effort made to ensure *Jitegemee* would be sustainable and observed that this is a key policy consideration in deciding whether to adopt a new intervention. They endorsed the intervention as designed in workshops 1 and 2, but recommended excluding NHIF because the government was in the process phasing it out, therefore its future was unknown at the time.

#### Final intervention design

**Fig. 1:**
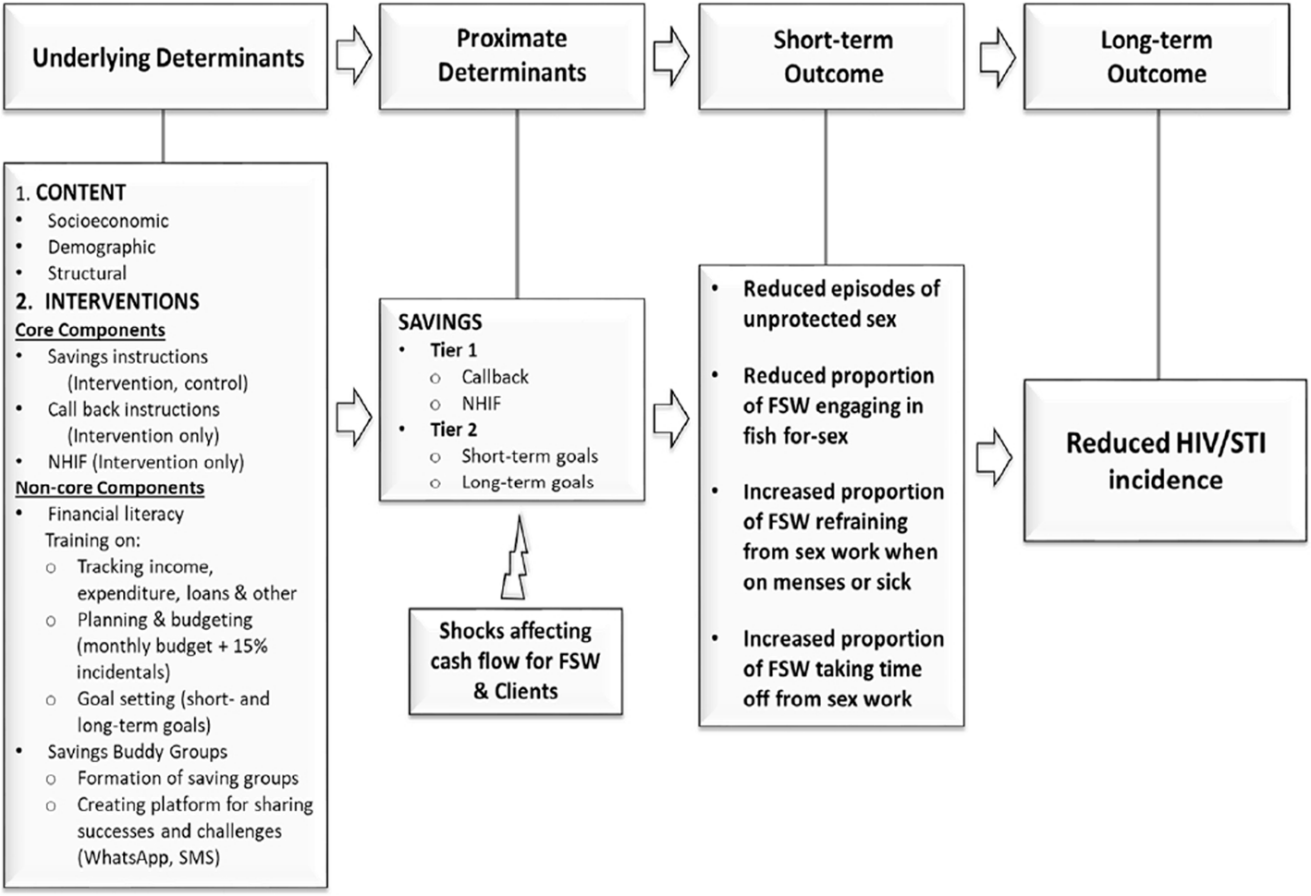
Intervention Model

**Fig. 2:**
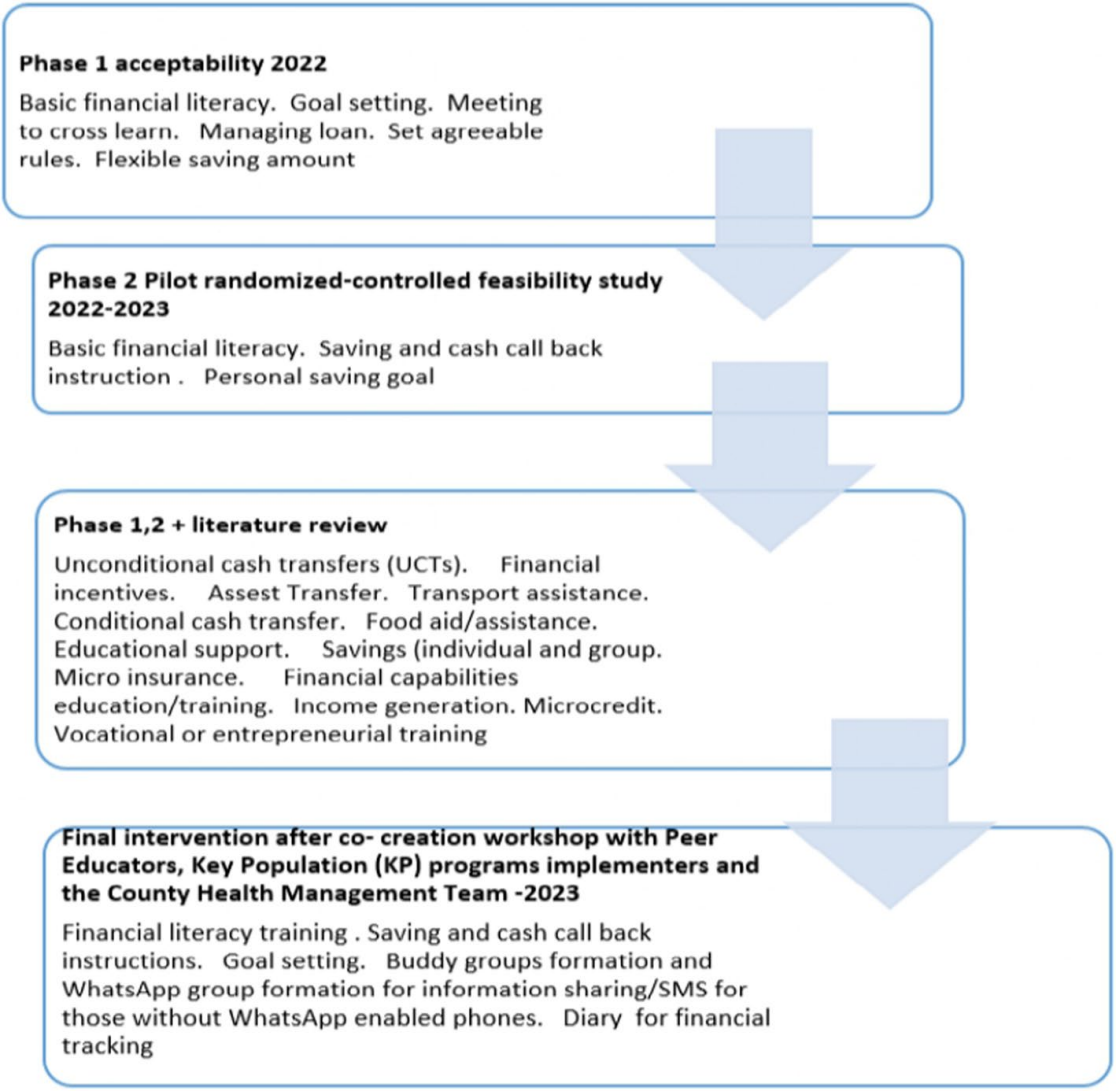
Jitegemee intervention development roadmap

The study team put all the recommendations together to arrive at the final intervention design, which we developed into a conceptual framework to demonstrate how the intervention is likely to bring change (Figs. 1 and 2). The final design is currently being piloted in a cluster randomized controlled trial in Kisumu County. It is comprised of core (mandatory) components that include savings instructions (given to both intervention and control participants) and callback, i.e. withdrawal, instructions (given to intervention participants only); and non-core (optional) components that include financial literacy and membership to a saving buddy group (intervention only).

## Discussion

This paper provides highlights of the process of co-creating a savings intervention to build financial reserves that FSW can draw from when faced with financial emergencies that might drive them to more risky sexual encounters. We adopted a PAR [22] approach and engaged representatives of FSW, a civil society organization overseeing the welfare of sex workers in western Kenya, key population program implementers, and policy makers and enforcers from the MOH to co-create the intervention. PAR has been recommended as a useful methodology for the purpose of facilitating empowerment given that it prioritizes the expertise of those experiencing a social issue (intervention beneficiaries) and uses systematic research methodologies to generate new insights [22]. This approach was instrumental in guiding our co-creation activities as we developed the *Jitegemee* intervention.

Through the co-creation process, our study resulted to the conceptualization of a saving-led intervention where FSW would save part of their income and call it back when faced with a financial need that could compel them to engage in HIV risk behaviours. Even though the choice of savings method depends on individual circumstances, financial goals, and personal preferences, this strategy was agreeable perhaps due to its comparative advantage. It presented a savings platform, namely as Mpesa, that requires a “soft commitment” in terms of restricting or charging minimal fees for withdrawals. Most people consider saving through insurance to be risky and uncertain [53] and requires a substantial fixed monthly premium. Saving in Rotating and Savings Credit Association (ROSCA) or self-help groups enables members to get loans with low interest rates along with easy and flexible repayment that could be sensitive to FSW’s social and financial circumstances. However, it involves opportunity cost of time spent attending meetings and the risk of default by members which has led to many such associations breaking [54]. On the other hand, saving money in the bank could be a safe alternative, possibly pay interest and can be linked to other services. However, most low income earning people are less attracted to save in banks due to the perceived bureaucratic processes and higher service fees.

Tracking income, expenditure, and loan-taking and –repayment were the three most important and sustainable components suggested by most participants in Phase 1 and selected during the intervention design development. As found by our study [41] and others in SSA [14, 55] and elsewhere [56], FSW generally believe they earn less than they actually do and most are unable to account for how they use their income [57], mainly because of impulsive buying [58]. FSW have peer-influenced substantial meaning to the idea of ‘quick money does not stay, which reflects their daily financial strategies [58]. Due to this mind-set and perhaps because of unplanned purchases and other financial burdens (the majority of our participants in Phase 1 reported being heads of their households and taking care of children), many FSW spend more than they earn, a findings that has also been in reported in Uganda [59] and Côte d’Ivoire [14] and which may explain their inability to save and invest in alternative sources of income or asset ownership). To fill this gap, FSW reported resorting to taking loans from friends, family and table banking groups (Ochieng’ LA, Onyango J, Owuor GO, Obare I, Bukusi E, Agot K: Saving for a rainy day: A randomized-controlled trial to pilot a savings intervention to reduce HIV risk among young female sex workers in Siaya County, Kenya, submitted), which adds to their financial burden. Studies have shown that cash expenses represent most of FSW spending, that their income is erratic from week to week, and that savings are regularly withdrawn [60–62]. Including the elements of income, expenditures, and loan management in the *Jitegemee* intervention would therefore address major reasons why most FSW are unable to become financially independent.

One of the key observations during Phase 2 pilot study was the marked reduction in saving and increase in withdrawals in 2 of the 6 months of follow up (Ochieng’ LA, Onyango J, Owuor GO, Obare I, Bukusi E, Agot K: Saving for a rainy day: A randomized-controlled trial to pilot a savings intervention to reduce HIV risk among young female sex workers in Siaya County, Kenya, submitted). We realized that the two months coincided with the periods when the State Department of Fisheries banned fishing to allow for reproduction. Given that many participants had clients who are fishermen, this shock reduced participants’ income from transactional sex and consequently, their savings as well. Anticipating, monitoring and preparing intervention recipients to manage economic shocks when they occur was deemed important in a savings intervention like *Jitegemee,* and participants advised intervention designers to consider factors that could undermine FSW’s ability to save, including inflation, slow client flow, and political unrest.

Our findings in this phase also showed that condom use was significantly higher among FSW who saved and withdrew their money at least once consistently used condom during transactional sex compared to those who saved but did not withdraw their money. Other studies on savings-led interventions among FSW have reported similar findings. In Iringa, Tanzania, FSW who participated in community savings groups, called *michezo*, were found to have increased odds of consistent condom use with new clients, compared to those who not exposed to the intervention [12]. In their study in Karnataka, India, found out that group saving and access to savings enabled FSW to refuse unsafe sex [47]. Similarly, the Pragati programme in India, which included a co-operative bank structure that provided a savings and credit mechanism, had a positive effect on condom use and STI incidence among FSW [48, 63].

The stakeholders who participated in the intervention design workshop (phase 5) pointed out that besides addressing structural, behavioral and biomedical determinants of HIV risk among FSW, the proposed savings intervention should also address sexual and gender-based violence. It has been observed that the link between economic empowerment and the risk of sexual and gender-based violence is theoretically ambiguous [64]. While it is generally touted as a potential means to reduce gender-based violence (GBV) against women [36, 65–67], it can exacerbate violence against FSW by clients [65] or third parties who may use intimidation or violence to force them to have unprotected sex. Our results therefore emphasize a need to prioritize women's safety in the process of designing economic empowerment programs that facilitate FSW’s ability to negotiate their risk environment in safer sex-work settings and more actively mitigate abuse and harassment by clients and third parties.

Developing an economic empowerment intervention for FSW should be based on their needs and perspectives as the beneficiaries in order to get unique insight into how good practice sex worker-led initiatives could be the benchmark for designing economic empowerment programmes in developing countries. Our findings provide evidence that the proposed *Jitegemee* intervention, is a feasible economic empowerment for FSW and can be implemented in other resource-limited counties. First, a key feature of *Jitegemee* is sustainability, hence the need to design an intervention that would not rely on external funding sources as most projects and programs that are externally funded, especially in developing countries, often fizzle out when funding ends [68–70]. In our study, the top-scored components were considered unsustainable, hence excluded. Secondly, the *Jitegemee* intervention is anchored on concept of mobile banking. There has been a notable increase in ownership of mobile phones and other digital devices in SSA over the decades [71, 72]. Implementing a savings-led economic empowerment intervention such as the *Jitegemee* intervention in other countries with similar socio-economic characteristics would be feasible. Thirdly, with limited literacy/ numeracy skills, as it is typical among most FSW in developing countries, it practical to use of simple diaries and equipping FSW with knowledge and skills on how to balance earnings, spending, savings, and taking and repaying loans as suggested by our participants.

### Limitations

The elaborate design process had some limitations. All the phases were conducted in three counties of western Kenya, thus the intervention may not be wholly applicable to FSW in the other 44 counties in Kenya. However, ensuring that we included key typologies of sex workers, a civil society organization for FSW, HIV program implementers with FSW, and MOH leadership overseeing HIV programming with FSW in their respective counties ensured that we based the design on a wide range of expertise that would make it easy to adopt or adapt the intervention to other settings. Even though NHIF was suggested so peers can save for emergency health needs, we eliminated it because we learned the country was on the verge of changing the system. We cannot be sure how the omission of this component will impact the overall intervention effectiveness.

The intervention development process has several strengths, most notably the multi-phase co-creation of the intervention, using PAR with potential end-users as well as other key stakeholders. We have also based the design on actual studies done among FSW by our team and in multiple settings in SSA and Asia, making the intervention more likely to be relevant beyond the study locations. Finally, in phase 2 we tested if FSW can save by giving them saving instructions and nothing else, and a sizable proportion were able to save. Adding the components obtained from FSW in phases 1 and 2, having FSW score and rank the components in order of preference, and engaging PE and civil society organization of FSW, as well as implementers of FSW programs and policy makers to co-create the intervention with the study team, will likely make *Jitegemee* more robust, sustainable, and therefore more effective in facilitating savings and reducing HIV risk among FSW.

## Conclusion

*Jitegemee* intervention was highly acceptable and feasible, and is potentially sustainable. Involving end-users and other key stakeholders in the design process will likely make the intervention more acceptable to FSW, encourage them to save more, and ensure financial security, thus reducing sexual encounters associated with higher HIV risk.

## Data Availability

All data generated or analysed during this study are included in this published article.

## Abbreviations

FSW: Female sex workers
UNAIDS: Joint United Nations Programme on HIV/AIDS
SSA: Sub-Saharan Africa
PAR: Participatory action research
MOH: Ministry of Health
PE: Peer educators
STI: Sexually transmitted infection
FGD: Focus group discussions
NHIF: National Hospital Insurance Fund
